# Functions and computational principles of serotonergic and related systems at multiple scales

**DOI:** 10.3389/fnint.2014.00023

**Published:** 2014-03-07

**Authors:** Kae Nakamura, KongFatt Wong-Lin

**Affiliations:** ^1^Department of Physiology, Kansai Medical UniversityOsaka, Japan; ^2^Intelligent Systems Research Centre, School of Computing and Intelligent Systems, University of UlsterNorthern Ireland, L'Derry, UK

**Keywords:** serotonin 5-HT, neural circuit, computational modeling, dopamine, serotonin, dorsal raphe nucleus, locus coeruleus, pendunculopontine tegmental nucleus

As one of the phylogenetically and ontogenetically oldest neurotransmitters, the monoamine serotonin (5-HT) is derived from tryptophan in neurons within the raphe nuclei, and innervates various parts of the nervous system (Jacobs and Azmitia, 1992). The serotonergic system is complex and can generate multifarious actions (Barnes and Sharp, 1999; Smythies, 2005). There are seven general families of serotonin receptors with multiple receptor subtypes, all of which are G protein-coupled receptors (GPCRs) except one (5-HT3 receptor), which is a ligand-gated ion channel, and these receptors can modulate the release of many major neurotransmitters such as glutamate, GABA, dopamine, acetylcholine, and norepinephrine (Barnes and Sharp, 1999; Smythies, 2005). It can also modulate neuronal excitability and network properties of many targeted brain areas, and regulate mood, cognition and behavior (Smythies, 2005). Dysfunctions of the serotonergic system are implicated in neuropsychiatric disorders including depression and schizophrenia (Müller and Jacobs, 2009). The serotonergic system has been the target of pharmaceuticals for decades, primarily to treat biological and neuropsychiatric disorders. These include antidepressants, antipsychotics, hallucinogens, antimigraine agents, and gastroprokinetic agents (Nichols and Nichols, 2008). Hence, the study of serotonin has high societal impacts.

Although the serotonergic system has been studied for many years, an integrative account of its underlying functions remains elusive. This could be partly attributed to the high variability and heterogeneity in terms of neuronal properties and receptor subtypes, and its extensive connections with other brain regions. Indeed, it has been claimed that serotonin is in involved “in virtually everything, but responsible for nothing” (Jacobs and Fornal, 1995). While there have already been many excellent reviews and books on serotonin and related neural systems (e.g., Jacobs and Azmitia, 1992; Barnes and Sharp, 1999; Smythies, 2005; Müller and Jacobs, 2009), we hope that this collection of recent works provides a complementary and updated coverage of their diverse functions. In particular, unlike previous collections, neurobiologically based computational studies are included in this collection as we consider them to be important toward elucidating some of the underlying principles, especially at the systems level. Hence, we have made a concerted effort to invite both experimental and computational articles in this Research Topic. These works include original results, reviews, and hypothesis over multiple levels: from receptors and channels, to neuronal circuits and finally to behavior and neuropsychiatric disorders.

At the receptor and cellular levels, Maejima et al. (2013) discussed various GPCRs and ion channels in the serotonin regulation and introduced optogenetic techniques that modulate intracellular signaling to more finely control the serotonergic systems for studies of their functions. The activation of the serotonin receptors was determined by its release and uptake dynamics. Unlike other more commonly studied neurotransmitters such as acetylcholine for example, the release and uptake dynamics of serotonin is not well characterized. Dankowski and Wightman (2013) reviewed the challenges and developments of fast-scan cyclic voltammetry to monitor serotonin at the subsecond (maybe millisecond) timescale in both *in vitro* and *in vivo* conditions.

At the neuronal circuit level, Celada et al., 2013 provided a comprehensive review on cortical modulation of serotonin. In particular, the prefrontal cortex, linked to executive brain functions, seemed to form closed-loop interactions with the serotonin neurons in the dorsal raphe nucleus. This review was well complemented by biologically realistic computational modeling works of serotonin modulation on the prefrontal cortex. In Wang and Wong-Lin (2013), a biologically motivated model was developed to investigate how the co-modulation of serotonin and dopamine in the prefrontal cortex could result in complex, non-intuitive neuronal circuit dynamics, thus challenging current simpler theories on neuromodulation. Cano-Colino et al. (2013) incorporated serotonin modulation into an established computational model of the prefrontal cortex performing spatial working memory tasks. The model showed that excessive serotonin could impede task performance, and interestingly predicted that serotonin levels could affect neuronal memory fields.

Besides the cortex, serotonin is also known to modulate important subcortical brain regions. Using a mathematical model of multiple brain regions, Reed et al. (2013) demonstrated the potential roles of serotonin in maintaining homeostasis in the basal ganglia (via the frontal cortex) under dopamine depletion (e.g., in Parkinson's disease). In Nakamura (2013), the neural circuit architecture of the dorsal raphe nucleus and other key subcortical brain regions involved in reward-based decision making and learning were discussed with emphasis on the neural circuit. The dorsal raphe nucleus has strong anatomical and functional connectivity with neighboring structures including the pendunculopontine tegmental nucleus (PPTg) and the locus coeruleus (LC), where many acetylcholine and noradrenergic neurons are found, respectively, (Koyama and Koyama, 1993; Martinez-Gonzalez et al., 2011). Indeed, Okada and Kobayashi (2013) showed that PPTg neurons exhibit similar tracking of future reward expectation as neurons in the dorsal raphe nucleus. Tsuruoka et al. (2012) reviewed the role of LC on pain control, which might be involved in aversive information processing.

It has been proposed that reinforcement learning models can be used as a platform for studying neurological and neuropsychiatric disorders (Maia and Frank, 2011). In this collection, Herzallah et al. (2013) dissociated among depressed patients with and without antidepressant medication, and healthy control subjects by observing the performance in learning from positive (reward) and negative (punishment) feedback. Castro-Rodrigues and Oliveira-Maia (2013) provided a useful commentary on this important original work. Finally, the comprehensive review by Asher et al. (2013) proposed a closed-loop paradigm toward understanding serotonergic roles in decision making by involving behavioral experiments, game theory, computational modeling, and human–robotic interaction, a truly integrative neuroscience approach.

We hope that this issue will provide a comprehensive review of the diverse and complex functions and computations of serotonergic and related systems at multiple scales of investigation. We wish that this will motivate and inspire a more integrative research approach from cellular to systems level toward understanding neuromodulatory systems.
